# Closing Editorial for Smart Healthcare: Technologies and Applications

**DOI:** 10.3390/healthcare12232363

**Published:** 2024-11-25

**Authors:** Gang Kou, Shuai Ding, Li Luo, Tian Lu, Yogesan Kanagasingam

**Affiliations:** 1 Xiangjiang Laboratory, Changsha 410205, China; kougang@swufe.edu.cn; 2 Big Data Laboratory on Financial Security and Behavior, SWUFE (Laboratory of Philosophy and Social Sciences, Ministry of Education), Chengdu 611130, China; 3 School of Management, Hefei University of Technology, Hefei 230009, China; 4 Business School, Sichuan University, Chengdu 610065, China; luolicc@scu.edu.cn; 5 W. P. Carey School of Business, Arizona State University, Tempe, AZ 85281, USA; lutian@asu.edu; 6 School of Medicine, The University of Notre Dame, Fremantle, WA 6160, Australia; yogi.kanagasingam@nd.edu.au; 7 St. John of God Midland Private and Public Hospitals, Midland, WA 6056, Australia; 8 Department of Health Care Policy, Harvard University, Cambridge, MA 02138, USA

It is our honor to present this editorial to close the Special Issue “Smart Healthcare: Technologies and Applications”, which we are coordinating.

“Smart Healthcare” refers to the utilization of next-generation information technologies to achieve personalized, intelligent, and interconnected healthcare services. With the increasing speed of emerging technologies, providing high-quality, high-quantity healthcare services to individuals has become much easier. Recent years have seen explosive growth in individual smart healthcare systems that can “connect people, materials, and institutions” and achieve the “active management” of medical ecosystems [1,2]. Yet, the vision of interconnected, whole-cycle healthcare requires the design of smart healthcare systems grounded in systems engineering and operations management theories. Unique solutions need to be developed for each component of the healthcare systems for them to become interconnected. These unique challenges in smart healthcare make it a highly interdisciplinary field, requiring knowledge from medicine, social sciences, bioinformatics, information science, systems engineering, and management sciences, among other things [3]. Therefore, we invited authors to publish their latest research related to the connectedness and whole-cycle application of smart healthcare, not limited to the topic of smart healthcare data governance, knowledge inference, smart healthcare systems engineering and operation management, etc. This Special Issue brings together 20 articles from 84 submissions, all exhibiting such interdisciplinary characteristics. Three types of articles can be observed amongst these contributions.

**(1)**  Novel Hardware for Smart Healthcare Applications

The first type of article mainly revolves around the utilization of new hardware to solve existing medical problems. Smart healthcare can involve the significant utilization of newly designed hardware, such as robotic surgery, yet could also be as unexpected as novel visual test equipment. Existing medical problems can be resolved more safely and more efficiently with such new technologies and their applications.

Abu Rass et al. (Contribution 1) compared the effectiveness of three audio guidance modalities (pitch sonification, verbal, and vibration) for helping visually impaired individuals locate a small cube in 3D space. While vibration and verbal guidance resulted in similar times and path lengths, sonification took significantly longer. Interestingly, despite the slower time, all participants reported similar levels of satisfaction with each method. The findings suggest that while sonification may not be the most time-efficient, verbal guidance might be the optimal choice for guiding visually impaired individuals in object location tasks.

Ben-Eli et al. (Contribution 4) evaluated the accuracy and reliability of a new self-administered near-visual acuity (VA) test (HSVA) compared to traditional Snellen and Rosenbaum charts. The HSVA showed strong test–retest reliability and accuracy that was comparable to examiner-administered tests. The study included 275 participants, demonstrating that the HSVA were able to accurately predict VA results obtained using standard clinical methods. The HSVA shows promise as a home-based tool for self-monitoring VA, particularly for individuals with chronic eye conditions or limited access to traditional eye care. Further research is warranted to explore its application in primary care, emergency medicine, and neurology.

Kim et al. (Contribution 12) presents a novel virtual-reality-based simulator designed for practicing vein blood sampling techniques, addressing limitations in traditional clinical practice environments. The proposed simulator utilizes a 3D model that simulates the vein blood sampling process, allowing users to engage through a head-mounted display (HMD) and haptic devices, facilitating a realistic training environment without physical constraints. Trainees can interact with a virtual patient model, enhancing their learning experience and skill acquisition. The simulator was tested for effectiveness among dental students, demonstrating its potential to provide comprehensive training in blood sampling techniques. The findings suggest that this innovative approach can significantly improve educational experiences in medical training by offering a safe, immersive, and flexible platform for practicing essential clinical skills.

Lei et al. (Contribution 14) combines low-pass whole genome sequencing and NGS-based STR tests to analyze miscarriage samples, significantly increasing the detection rate of chromosomal abnormalities to 56.4% in 500 cases. This novel system, unlike traditional methods, can identify triploidy, uniparental diploidy, maternal cell contamination, and the parental origin of erroneous chromosomes. Trisomy was the most frequent aneuploidy, with 94.7% of extra chromosomes originating from the mother. This enhanced genetic analysis method provides valuable insights for clinical pregnancy guidance.

Rêgo et al. (Contribution 18) evaluates the physical and comfort qualities of two clothing prototypes incorporating sensors for pressure, temperature, and humidity, aiming to prevent pressure injuries. Nine expert nurses assessed the prototypes using questionnaires and focus groups. Both prototypes received low scores for stiffness and roughness, with Prototype A receiving the lowest score for stiffness. Participants highlighted comfort concerns and suggested improvements. The study concludes that the prototypes need significant improvements in stiffness and roughness to enhance their safety and comfort for users, potentially hindering their practicality for pressure injury prevention.

**(2)**  Novel Algorithms on Existing Smart Healthcare Infrastructure

The second trend is, in contrast with the first trend, the utilization of novel, carefully designed information systems to address current healthcare challenges. Instead of introducing new materials, existing infrastructure, such as smartphone sensors, environmental technologies, cameras, internet, Internet of Medical Things, and existing information systems can be modified and augmented to serve smart healthcare purposes. 

Baptista et al. (Contribution 2) examine pharmacy interventions (PIs) in a community hospital over a period of 8 months, highlighting the crucial role of pharmacists in patient safety. Using the xPIRT tool, the researchers identified numerous prescribing errors, highlighting potential discrepancies between community and acute hospital settings. The analysis revealed a need for integrated electronic prescribing systems combined with PI recording tools for enhanced accuracy. The study emphasizes the significant contribution of pharmacy teams to patient safety, care quality, and cost savings through effective PI implementation.

Beldjoudi et al. (Contribution 3) present an oncology data management (ODM) tool, developed for radiotherapy, which extracts data from an oncology information system (OIS) to create structured information used for monitoring and improving patient care. By analyzing data from 2016–2022, ODM revealed discrepancies in treatment machine utilization and identified opportunities for quality improvement. The introduction of ODM led to the implementation of quality indicators and organizational changes, resulting in shorter treatment preparation times for palliative patients and decreased prescription variability in breast cancer treatments. Overall, ODM demonstrates its potential to enhance radiotherapy services by providing quantitative insights for optimizing workflow and improving patient outcomes.

Di Fazio et al. (Contribution 6) propose a telematic approach for social workers on medico-legal commissions of Italian National Institute of Social Welfare (INPS) to assess disability status remotely. This method aims to improve accessibility and efficiency by eliminating the need for in-person visits, particularly for individuals who cannot be physically moved or when document evaluation is sufficient. The proposed protocol involves conducting a remote session via platforms like Skype to gather information about the patient’s socio-environmental situation. While recognizing that telemedicine cannot completely replace face-to-face interactions, this approach offers an alternative to optimize the disability assessment process, reducing travel time and relying on more than just phone calls.

Ellington et al. (Contribution 7) investigate the potential of speckle plethysmography (SPG) as a signal for continuous non-invasive blood pressure (CNBP) monitoring using pulse arrival time (PAT). Comparing SPG with photoplethysmography (PPG), the study found that SPG, paired with ECG, provides comparable or even better performance in predicting systolic blood pressure (SBP) using machine learning models (support vector regression and decision tree regression). Both SPG and PPG showed reduced prediction errors when incorporating the previous SBP value. The study concludes that SPG holds significant promise for CNBP monitoring, urging further investigation with larger cohorts and improved SPG acquisition systems.

Fiani et al. (Contribution 9) investigate the effectiveness of virtual interfaces for remote psychotherapy, specifically focusing on a mindfulness meditation protocol. The researchers combined machine learning techniques for distance detection, camera calibration, and eye tracking to create a virtual environment for the protocol, demonstrating that participants achieved a higher level of concentration and lower stress compared to a control task. This preliminary success suggests that virtual psychotherapy can produce results comparable to in-person sessions, opening doors for further research and the development of a fully functional interface for remote applications, particularly where therapist presence is limited.

Hosseini et al. (Contribution 11) review the impact of indoor lighting conditions on health outcomes in healthcare environments, drawing on neuroscience and biological evidence that underscores the significance of natural light. The objective is to assess existing scientific studies to inform lighting designs that support circadian rhythms. A systematic methodology was employed, comprising literature searches, screening, and appraisal to evaluate the relevance and quality of the studies. Through thematic analysis, findings were categorized into two groups: interventional studies involving human subjects and simulation-based studies. While there is strong evidence linking natural light to improved health and well-being, the review indicates that the benefits of artificial lighting in healthcare settings are less clear, attributed to inconsistent lighting designs, varied implementation of interventions, and differing study populations. The authors recommend future research to adopt standardized metrics and methods to bridge theoretical insights with practical applications in healthcare lighting design. They advocate for collaboration among architects, designers, lighting experts, and healthcare professionals to enhance the evidence base for effective artificial lighting strategies in these environments.

Lim et al. (Contribution 15) compare the diagnostic yield and complication rates of frameless navigation-guided brain biopsy with frame-based stereotactic biopsy. Results from 42 frameless biopsies showed a 100% diagnostic yield, with only 2.4% of patients experiencing asymptomatic intracerebral hematoma. This compares favorably to frame-based biopsy, which had a 96.7% diagnostic yield and no significant difference in complication rates. The authors conclude that frameless navigation-guided biopsy is as effective and safe as frame-based biopsy, suggesting it may be a preferred method. However, they acknowledge the need for further research to validate these findings.

Perlea et al. (Contribution 17) present a new, simplified digital workflow for creating hybrid posts and cores in-office using CAD-CAM technology. The method involves scanning the prepared tooth with a specific bur placed within the canal, followed by a second scan without the bur. This “Pre-Preparation” scan facilitates the accurate design of the post and core within the software. The procedure eliminates the need for conventional impressions, allowing for the same-day delivery of the hybrid post and core. The authors highlight the advantages of using zirconia and fiber posts for anterior tooth restoration, emphasizing their biomechanical properties and esthetic benefits.

Shakya et al. (Contribution 19) explore the use of image compression techniques combined with statistical texture analysis to optimize the storage of DICOM files, particularly in the context of COVID-19. The research evaluated four compression algorithms (DCT, DWT, FCA, VQA) for their ability to compress data while preserving essential texture features. The findings reveal that despite achieving varying compression ratios, all algorithms successfully maintained critical texture information. The study also highlights the effectiveness of GLCM in analyzing texture changes in CT scans of COVID-19 patients. Overall, this research suggests that combining image compression with texture analysis offers a promising approach for the efficient storage and improved diagnostic assessment of medical images in a resource-constrained environment.

Zandonà et al. (Contribution 20) aim to improve the accuracy of trans-perineal prostate biopsies by comparing different kinematic models for robot-assisted needle insertion. The study focuses on the interaction between a bevel-tip needle and tissue, taking into account tissue heterogeneity, needle bending, and tissue deformation. The researchers conducted experiments using automated needle insertions into silicone phantoms under stereo-image guidance, comparing the accuracy of existing models in predicting needle deformation across varying speeds and tissue stiffness levels. Ultimately, this study aims to identify the most accurate model for predicting needle behavior, paving the way for more precise robotic needle insertion during prostate biopsies.

**(3)**  Empirical Analysis of Smart Healthcare Systems

At the center of these hardware and information system innovations stand managerial insights and investigations into current smart healthcare systems. Empirical analysis of recent smart healthcare applications presents new routes for smart healthcare system improvements and exposes potential pitfalls for such systems. On the other hand, operation management methodologies could be applied to increase the efficiency and safety of current smart healthcare systems.

Cammisuli et al. (Contribution 5) created a study protocol that investigates spatial navigation (SN) in 76 older adults: healthy controls, individuals with subjective cognitive decline (SCD), and patients with mild cognitive impairment (MCI) due to Alzheimer’s disease (AD). Participants will complete a virtual (AppleGame) and naturalistic (Detour Navigation Test) SN tasks. The authors aim to: (1) assess SN performance in each group, focusing on the impact of APOE-ε4 carrier status; (2) determine if virtual task performance predicts ecological performance; and (3) explore the potential use of SN assessments in early AD detection and rehabilitation. The researchers hypothesize that MCI due to AD patients, especially APOE-ε4 carriers, will exhibit impaired SN compared to SCD and healthy controls. The study hopes to improve our understanding of SN decline in AD and inform the development of interventions to mitigate spatial disorientation in older adults.

Feroz et al. (Contribution 8) explore the perspectives of caregivers in Pakistan on a mobile phone-based telemonitoring (TM) program for pregnant women at risk of pre-eclampsia. Caregivers overwhelmingly welcomed the program, citing benefits like reduced anxiety and workload, increased convenience, and cost-effectiveness. However, concerns arose about program accessibility for women and caregivers with limited technological skills and low literacy. The study emphasizes the need for: (1) a user-centric design tailored to the local context; (2) comprehensive caregiver training; (3) promotion of the program’s benefits; and (4) making the program affordable to maximize access. These findings provide valuable insights for developing effective and culturally sensitive TM interventions in low–middle-income countries.

He et al. (Contribution 10) address the challenge of optimizing large-scale COVID-19 nucleic acid testing through a dynamic testing site deployment strategy. A multi-period location–allocation model was developed to account for the spatial-temporal distribution of the testing population and the fluctuating availability of testing resources. The model was compared against several static site deployment strategies to demonstrate its advantages. A real-world case study in the Chenghua district of Chengdu, China, showed that the dynamic strategy could reduce overall costs by 15% compared to an actual implementation and about 2% less than other static models. Additionally, sensitivity analysis provided insights for enhancing site deployment practices. The findings emphasize the significance of adapting testing site locations based on population dynamics to lower costs and improve operational efficiency amidst limited resources.

Koeck et al. (Contribution 13) investigate the occurrence of drug-related problems (DRPs) in telepharmacy consultations for ICU patients, both with and without COVID-19, within a German telemedicine network. The analysis found that patients with acute renal insufficiency and without renal replacement therapy had a significantly higher risk of DRPs compared to those with normal renal function, regardless of COVID-19 status. Notably, COVID-19 patients who received therapeutic anticoagulation (Heparin group) exhibited significantly more DRPs than non-COVID-19 patients. The researchers suggest prioritizing telepharmacy consultations for COVID-19 patients receiving therapeutic anticoagulation and all ICU patients with renal insufficiency, highlighting the importance of telepharmacy in mitigating DRPs during complex healthcare scenarios.

Mpagama et al. (Contribution 16) tested the feasibility, acceptability, and effectiveness of an adaptive diseases control expert program (ADEPT) in Tanzania, using TB and DM management as a prototype. The ADEPT involved a stepwise training approach with web-based platforms and a clinical audit. Results showed that ADEPT-intervention facilities detected significantly more individuals with dual TB and DM compared to control facilities. The model led to the higher use of HbA1c testing in intervention facilities. While training was successful, differences in screening for other comorbidities were not observed. The study concludes that the ADEPT model is feasible, acceptable, and effective in Tanzania, potentially improving the quality of healthcare services when adapted to the local context.

**(4)**  Future Perspectives

The constant development of new technologies generates highly sophisticated smart healthcare systems each year. From the submissions on this topic, we have observed a trend of individual systems being scattered across various healthcare fields, such as diagnosis, surgery, and elder care. However, to realize the vision of “personalized, intelligent, and interconnected” healthcare services, these individual systems and innovations must be integrated into a cohesive framework. Some of the articles in this collection illustrate this necessity, such as Contribution 11, which connects hospital environments to personalized healthcare, and Contributions 9 and 12, which combine psychological treatment with physiological training using virtual reality (VR). Ultimately, the interconnectedness of individual smart healthcare systems must be achieved through systems engineering and the design of organic protocols for data utilization, governance, and information exchange. These protocols should enable all smart healthcare systems to adapt effectively. Given that healthcare is a critical industry, these designs must adhere to the principles of safety, privacy, and efficiency.

Recently developed technologies could facilitate the interconnectedness of smart healthcare systems. General-purpose AI models, including large language models (LLMs) with vision capabilities, are designed to collect, analyze, memorize, and understand complex scenarios with diverse designs while providing personalized and intelligent interactions with users. In this context, the utilization of such general-purpose models represents a significant breakthrough for the future development of smart healthcare system engineering and operations management. However, challenges still exist regarding the safety, privacy, and efficiency of these general-purpose LLMs, which could become a focal point for the next generation of smart healthcare system research.

Finally, as the topic editors of “Smart Healthcare: Technologies and Applications”, we would like to thank and acknowledge all the authors and reviewers of the manuscripts submitted to this Special Issue, as well as the editorial teams of the corresponding journals for their administrative support.
